# Molecular Mechanisms Driving IL-10- Producing B Cells Functions: STAT3 and c-MAF as Underestimated Central Key Regulators?

**DOI:** 10.3389/fimmu.2022.818814

**Published:** 2022-03-10

**Authors:** Magalie Michée-Cospolite, Marina Boudigou, Alexis Grasseau, Quentin Simon, Olivier Mignen, Jacques-Olivier Pers, Divi Cornec, Laëtitia Le Pottier, Sophie Hillion

**Affiliations:** ^1^ U1227, LBAI, Univ Brest, Inserm, Brest, France; ^2^ U1227, LBAI, Univ Brest, Inserm, and CHU Brest, Brest, France

**Keywords:** regulatory B cells, molecular drivers, c-MAF, STAT3, B-cell differentiation

## Abstract

Regulatory B cells (Bregs) have been highlighted in very different pathology settings including autoimmune diseases, allergy, graft rejection, and cancer. Improving tools for the characterization of Bregs has become the main objective especially in humans. Transitional, mature B cells and plasma cells can differentiate into IL-10 producing Bregs in both mice and humans, suggesting that Bregs are not derived from unique precursors but may arise from different competent progenitors at unrestricted development stages. Moreover, in addition to IL-10 production, regulatory B cells used a broad range of suppressing mechanisms to modulate the immune response. Although Bregs have been consistently described in the literature, only a few reports described the molecular aspects that control the acquisition of the regulatory function. In this manuscript, we detailed the latest reports describing the control of IL-10, TGFβ, and GZMB production in different Breg subsets at the molecular level. We focused on the understanding of the role of the transcription factors STAT3 and c-MAF in controlling IL-10 production in murine and human B cells and how these factors may represent an important crossroad of several key drivers of the Breg response. Finally, we provided original data supporting the evidence that MAF is expressed in human IL-10- producing plasmablast and could be induced *in vitro* following different stimulation cocktails. At steady state, we reported that MAF is expressed in specific human B-cell tonsillar subsets including the IgD^+^ CD27^+^ unswitched population, germinal center cells and plasmablast.

## Introduction

B cells with immunosuppressive function (Bregs) have been highlighted for the first time in the murine experimental autoimmune encephalomyelitis (EAE) model (1). Although the induction of the disease was not influenced by the absence of B cells, B-cell depleted mice exhibited no substantial recovery suggesting a regulatory role. During the past decade, various studies have reported this regulatory B cell-induced immune response suppression in very diverse pathophysiological settings like autoimmune diseases, transplantation, virus immunity, and cancer. The heterogeneity of the Breg subsets is a subject of interest for many years (2–5) and consensus opinions suggest that Bregs may arise from multiple progenitors depending on the microenvironment. Beyond their phenotypic status, Bregs used different immunosuppressive mechanisms such as the production of interleukin (IL)-10, granzyme B (GZMB), and transforming growth factor (TGF) β to regulate other immune cells (3, 6, 7). IL-10 has become the most documented suppressive mechanism by B cells and in many cases, IL-10 can be often co-expressed with other regulatory molecules such as TGFβ or IL-35 (8). New studies are now starting to clarify the different molecular mechanisms that may control the acquisition of the Breg function. In this review, we described the last reports establishing new insights into the molecular control of TGF β, GZMB, and IL-10 producing B cells (B10) with a specific focus of an understudied transcription factor (TF) in B cells: *the avian musculoaponeurotic fibrosarcoma oncogene* c-MAF (MAF).

## Molecular Control of the Production of TGFβ and GZMB in B Cells

### TGFβ -Producing Regulatory B Cells

TGFβ-producing B cells with regulatory properties were first described in cancer, especially in lung metastasis from breast cancer in mouse models (9). TGF-β^+^ Bregs that exhibited a particular phenotype (CD19^+^, CD25^high^, IgD^high^, CD21^low^, CD23^low^, CD43^-^, IgM^int^, and CD62L^low^) were able to decrease T cell proliferation and induce *de novo* regulatory T cells (Tregs) (Foxp3^+^) *in vivo* and *in vitro*. In this study, they demonstrated that the tumor-microenvironment was necessary to both mouse and human Breg development. These TGFβ-expressing Bregs expressed high levels of phosphorylated-STAT3 (pSTAT3). The same group further demonstrated that the inhibition of STAT3 phosphorylation by resveratrol (phytoalexin found in some plants as mulberries or peanuts) induced the decreased expression of TGFβ by Bregs leading to the reduced expansion of Tregs *in vivo* and *in vitro* (10). Inhibitory effect of the resveratrol as a potent inhibitor of STAT3 phosphorylation and acetylation is mostly mediated by the Sirtuin 1 protein (SIRT1) an NAD^+^-dependent class III histone deacetylase (HDAC). Although SIRT1/STAT3 axis has been extensively studied in cancer cells (11, 12), there are few reports on its role on B-cells. However, it was recently demonstrated that B-cell specific SIRT1 deficient cells displayed an increase of IL10 expression *in vitro* following LPS stimulation (13). Moreover, SIRT1 is highly expressed in resting naïve mouse B cells but dramatically reduced following cell activation and immunization. In this context, SIRT1 suppressed AICDA expression and the class switch DNA recombination through the regulation of the acetylation of STAT3 and the NFkB p65, another important target of SIRT1 (14). Underlying mechanisms of these different interactions with other molecular drivers of TGFβ are still unclear, especially in B cells.

Some studies highlighted the role of the different TGFβ isoforms in the regulatory properties of B cells. A first mouse study revealed the co-expression of TGFβ1 and TGFβ3 at similar levels in resting B cells but in IgM-activated B cells, TFGβ1 was increased and TGFβ3 was highly reduced. In a coculture model with resting B cells and T cells, Treg expansion was only dependent on the TGFβ3 isoform (15). However, in the EAE mouse model, TGFβ1-deficient B cells led to increased EAE score level and the disease severity was linked to the expansion of T cell helper (Th)1 in conserved non-coding sequences (CNS) (16). So, the two isoforms TGFβ1 and TGFβ3 seem to be implicated in Breg properties but with microenvironment-dependent mechanisms. Unlike in mice, the activation of human B cells upon BCR and Toll-like receptor (TLR) 9 induced decreased expression of TGFβ1 (highly expressed in resting B cells) but an increase of IL-10 expression (17). Unfortunately, in most of the studies on TGFβ-expressing Bregs, the nature of the isoform involved is still elusive.

The co-expression of TGFβ and IL-10 by Bregs is frequently reported in the literature but the role of TGFβ is controversial. One study demonstrated that B cells infiltrating tumors expressed TGFβ and IL-10 and inhibited T cell proliferation and the generation of interferon (IFN)γ-CD8^+^ T cells. This mechanism was TGFβ-dependent (18). However, in the model of low-dose methotrexate-induced tolerance in mice, the generated Bregs co-expressed TGFβ and IL-10, but only IL-10 seemed to be implicated in the tolerance mechanism (19). Two additional studies performed in humans demonstrated the role of TGFβ in Bregs-mediated regulation. In the first study, *in vitro* Bregs were induced following a CD40 ligand (CD40L), CpG, and IL-4 stimulation and were able to decrease T-cell proliferation and induce the Tregs expansion. FoxP3 expression and Tregs generation were reduced in the presence of the anti-TGFβ blocking antibody (Ab) but not after IL-10 blocking (6). We also demonstrated that *in vitro*-induced Bregs used a TGFβ-dependent mechanism in controlling T -cell proliferation whereas the decrease of IFNγ-Th1 cells was IL-10-dependent (20).

Other information puzzled the understanding of the generation of TGFβ-producing-Bregs because TGFβ was first described as able to inhibit B cell proliferation and Ig production (21). Moreover, a recent study highlighted the synergy between IL-10 and TGFβ3 to inhibit B-cell proliferation and Ig production after TLR4 or TLR7 activation by decreasing energy metabolism of B cells such as glycolysis. Interestingly, pSTAT3 was also expressed in these B cells (22). However, the synergistic role of IL-10 and TGFβ in controlling Bregs function remains to be explored.

### Expression of GZMB in B Cells

Initially, the GZMB had been described as a compound of the cytoplasmic granules of cytotoxic cells that induced targeted cell death when accompanied by perforin (23). However, over the last decade, the secretion of GZMB by B cells, Tregs, or plasmacytoïd dendritic cells, has been found to exert an immunosuppressive effect through performing-independent mechanisms. This GZMB-expressing Breg subset has been detected in a variety of pathological contexts: chronic lymphocytic leukemia (CLL) (24), solid tumor infiltration (7), autoimmune diseases such as Systemic Lupus Erythematosus (SLE) (25), viral infections (26) and in the context of the transplantation (27). Although GZMB-expressing B cells were found to be enriched in differentiated B cells like plasmablasts (PB), less than 3% of circulating B cells seemed to express GZMB in healthy individuals (28).

One of the key inducers of the production of GZMB in B cells is IL-21. Jahrsdörfer’s group first reported that IL-21 directly induced the secretion of enzymatically active GZMB by human B cells. The effect of IL-21 is synergically enhanced by CpG-ODN or BCR engagement (24, 29). Moreover, they demonstrated that GZMB and IL-21 serum levels are highly correlated in SLE and that CD5^+^ B cells from SLE and Sjögren syndrome patients, which constitutively expressed GZMB, displayed higher expression of IL-21 receptor (30). IL-21 is mainly secreted by activated CD4^+^ T cells, especially Th17 and follicular helper T cell (Tfh) subsets and natural killer T cell (NKT) (31). A study has shown that macrophages-derived IL-15 synergized with IL-21 to promote GZMB expression and B cell differentiation into CD38^+^ CD20^-^ PB. While IL-21 alone induced a quick and massive transcription of GZMB mRNA but a minimal production of the protein, the addition of IL-15 allowed efficient translation and/or secretion of the GZMB protein (32).

Little is known about the molecular control of GZMB production in B cells and the generation of GZMB^+^ Bregs remains to be understood. Besides, the inability of mouse B cells to secrete GZMB further complicates addressing this question (33). Nevertheless, it would appear that pSTAT3 signaling is implicated in GZMB expression. IL-21 signaling is known to activate JAK/STAT pathways, especially pSTAT1 and pSTAT3, and to a lesser extent pSTAT5a and pSTAT5b (34). An up-regulation of pJAK1, pJAK3, and pSTAT3, but not pTYK2 or pSTAT1, was observed in B cells stimulated with IL-21 and anti-BCR, and the use of a JAK inhibitor abrogated the GZMB production (29). The use of STAT1, STAT3, or STAT5 inhibitors confirmed the involvement of STAT3 signaling but not STAT1 and STAT5, in GZMB synthesis. Moreover, B cells failed to produce GZMB in patients with the autosomal-dominant hyper-IgE syndrome which exhibit heterozygous STAT3 mutations, confirming the unique role of STAT3 in GZMB production (32).

Recently, Sophie Brouard’s group has developed an expansion mixture containing IL-21, anti-BCR, CpG ODN, IL-2, and CD40L, to induce GZMB expression in more than 90% of B cells. While the presence of IL-21 and anti-BCR seemed indispensable to induce a strong expression of GZMB, the presence of CpG ODN and IL-2 seemed to be necessary for the maintaining of B-cell survival in culture (28). To better characterize GZMB-producing Bregs, they performed RNAseq analysis of sorted *in vitro*-expanded GZMB-positive and GZMB-negative B cells (35). Thirty-six genes were found to be differentially expressed between these two populations, including the top differential expressed genes (DEGs) *GZMB*, *LAG3*, and *FASLG*, which were already described for their role in immune regulation. They revealed a higher frequency of LAG-3^+^ cells but a similar expression of IL-10 in GZMB-positive Bregs when compared to GZMB-negative B cells. In this transcriptomic analysis, *SOX5* was the only gene encoding a TF found to be differentially expressed. While *SOX5* was found to be downregulated during the proliferation of B cells, its high expression might limit proliferation allowing PB differentiation (36). However, as an autocrine production in GZMB^+^ Bregs, the GZMB stimulates the proliferation of these cells (28). At this point, it is difficult to say whether the *SOX5* down-regulation may play a role in the generation and function of GZMB-positive Bregs or whether it is only a consequence of the autocrine production of GZMB itself.

## STAT3 and its partners in the generation of IL-10- producing B cells

IL-10 is a pleiotropic cytokine produced by virtually all immune cell types and possesses immunosuppressive properties. IL-10 is an inhibitor of a broad spectrum of monocytes/macrophages and T-cell functions, including cytokine synthesis, effector subset differentiation, and expression of co-stimulatory molecules such as CD80/CD86. However, the role of IL-10 on B cells was described as ambivalent. First reports described IL-10 as a growth factor for B cells. It promotes B-cell proliferation, Ab production, and class II MHC expression depending on their activation state (37). The addition of IL-10 in CD40-activated B cells results in a very high immunoglobulin production indicating differentiation of B cells into plasma cells (38, 39). IL-10 was an essential factor for isotype switching in presence of anti-CD40 stimulation leading to the production of IgM, IgG, and IgA (IgA1 and IgA2) in combination with TGFβ (40). Subsequent important reports described the role of IL-10 in promoting the differentiation of memory B cells into PB and plasma cells (41, 42).

**Figure 1:**
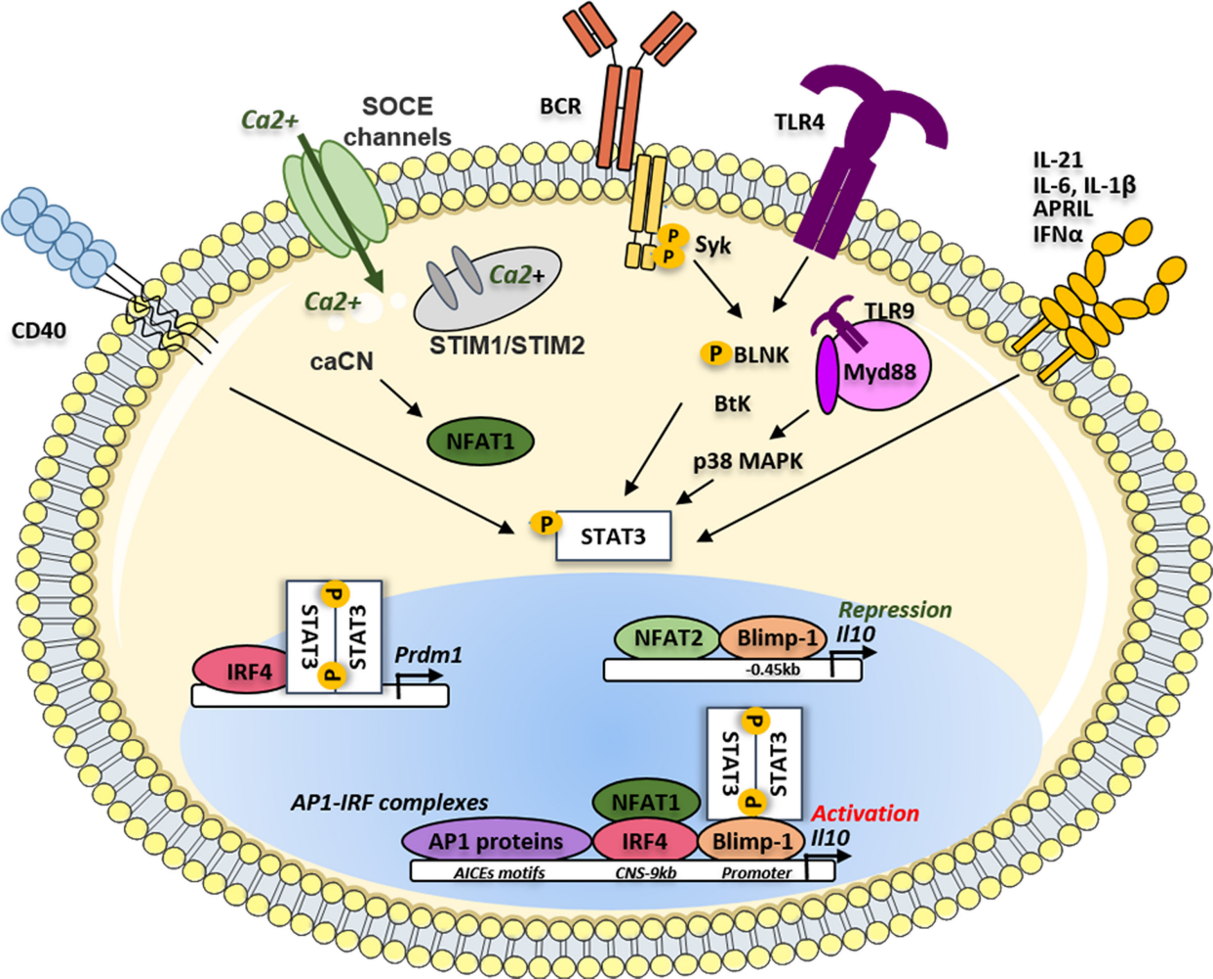
IRF4/Blimp-1/STAT3 activation pathways on *Il10* gene regulation in Bregs. The phosphorylation of STAT3 could be activated following different microenvironment signals such as different cytokines (APRIL, IL-21, IL-6, IL-1β and IFNα), the antigen or TLR ligands. Phosphorylated STAT3, translocates to the nucleus and could bind IRF4 to induce the transcription of the Prdm1 gene. The translation of Prdm1 gene leads to the production of the Blimp-1 protein supporting the binding of STAT3 on the *Il10* gene promoter. The binding of Blimp-1 together with NFAT2 could induce repression of the *Il10* gene transcription. The different AP1 proteins such as BATF, Jun B and Jun D could bind the AICES motifs on the Il10 gene to promote its transcription through the cooperation of IRF4. IL, Interleukin; IFNα, Interferon alpha; APRIL, A Proliferation inducing ligand; SOCE, Store operated calcium entry; TLR, toll-like receptor; CNS, conserved non-coding sequence; TLR, toll-like receptor BCR, B-cell receptor; BtK, Bruton tyrosine kinase; BLNK, B-cell linker protein; MAPK, Mitogen-activated protein kinase; Syk, Spleen associated tyrosine kinase; STAT3, Signal transducer and activator of transcription 3; NFAT, Nuclear factor of activated T-cells; STIM, Stromal interaction molecules; CaCN, active calcineurin A; Myd88, Myeloid differentiation primary response protein; Blimp-1, B lymphocyte-induced maturation protein 1; Prdm1, PR domain zinc finger protein 1; AP1, Activator protein 1; IRF, Interferon regulatory factor; AICEs, AP1-IRF-composite elements.

B cells secrete a very low amount of IL-10 at a quiescent state however specific stimulatory signals were described to be involved in the induction of IL-10 producing B cells with suppressive functions: the BCR, CD40, TLR, and cytokine signaling including IL-21, IL-6, or TGFβ (43–45). Although the time-sequence and location of these events have to be clarified *in vivo*, the molecular signals leading to IL-10 production by B cells have been studied less thoroughly than in Th cells and macrophages (reviewed in (46)). Among the key factors involved in the control of IL-10 production, STAT3 appears to be involved in the different activation cascades leading to IL-10 secretion by B cells (**Figure 1**). It was demonstrated in CD1d^hi^ CD5^+^ B10 cells, that whilst the absence of the B-cell linker protein (BLNK) had no consequence on the distribution of Bregs in tissues, its expression was indispensable to IL-10 production. After TLR4 activation by lipopolysaccharide (LPS), phosphorylated BLNK interacts with the Bruton tyrosine kinase (Btk) leading to the phosphorylation of STAT3 that next translocates to the nucleus to transactivate the *IL10* gene (47) (**Figure 1**). In SLE patients, CD24^high^ CD38^high^ Bregs exhibited a defect in CD40 signaling with impairment of STAT3 phosphorylation and IL-10 production (48). Similar results were described in patients with systemic sclerosis where TLR9-induced IL-10 production by B cells was impaired because of a defect in p38 MAPK and STAT3 signaling (49, 50). Various cytokines increased *in vitro* the generation of human B10, often in combination with TLR activation in a STAT3-dependent manner including APRIL (51), IFNα (52, 53), IL-35 (54), IL-21 (55), IL-6, or IL-1β (56). Interestingly mice with the specific deletion of STAT3 in B cells developed a severe experimental autoimmune uveitis and displayed exacerbated EAE (57). In both diseases, the deficiency of STAT3 in B cells induced a defect not only in the induction of B10 cells but also in the generation of Tregs. Moreover, the authors demonstrated that expression of the CD80 and CD86 costimulatory molecules was markedly upregulated on B cells in STAT3-deficient mice, suggesting that STAT3 signaling may also contribute to restraining excessive activation of T cells.

### Cooperation of BLIMP1 and IRF4 in the Generation of IL10 Producing B Cells

Once activated, STAT proteins can directly bind on a single motif on the *Il10* promoter (58) or may interact with other TF to induce IL-10 expression. The mouse *Il10* locus has been extensively studied in naive and differentiated T cells (59–61) mostly using DNase I hypersensitivity approaches highlighting at least nine genomic regions with potent high transcriptional activity. These several conserved noncoding sequences (CNS) presented a great homology between mice and humans. Together with the promoter, the CNS-9, CNS-4.5, located at 9 kb and 4.5 kb, respectively, upstream the *Il10* gene transcription start site (TSS), and the CNS+3, and CNS+6.5 Kb, located downstream the TSS, have been described as the main activator sites of the *Il10* transcription in T-cell subsets (62, 63). In Th1 cells, the B-lymphocyte-induced maturation protein 1 (Blimp-1) binds the CNS-9 site in a STAT4-dependent manner, and mice with a T-cell-specific Blimp-1 deficiency had a severe inflammatory response during *T. gondii* infection, suggesting that Blimp-1 was involved in the IL-10–dependent control of Th1 immune responses (64)

However, the role of Blimp-1 in the generation of B10 is conflicting. In a recent study, *Prdm1* mRNA expression was shown upregulated in IL10-positive compared to IL-10-negative B cells following LPS stimulation. This was then confirmed *in vivo* using a *Prdm1*-EYFP reporter mouse model. This group also demonstrated that the deletion of *Prdm1* led to the increased frequency of B10 cells following LPS injection suggesting a negative role of Blimp-1 on the *Il10* transcription. Using luciferase reporter assays and chromatin immunoprecipitation (ChIP), these authors demonstrated that the CNS-0.45 kb site on the *Il10* gene was the major Blimp-1 suppressive site of the *Il10* transcription (**Figure 1**). They further demonstrated that STAT3 needed the Blimp1 DNA-binding domain to efficiently transactivate the *Il10* promoter indicating that Blimp1 could exhibit alternatively a positive role and negative role depending on the presence or absence of its molecular partners (65). However, these binding sites were not reported in primary studies identifying the pattern of regulatory regions within the *Il10* gene in T cells suggesting a B cell-specific regulation of the *Il10* transcription (46, 59, 61).

The Blimp-1 regulated genes have been extensively studied during the B-cell differentiation, revealing that Blimp1 could act as both transcriptional repressor and activator. Using streptavidin-mediated chromatin precipitation coupled with deep sequencing (Bio-ChIP), the *Il10* gene was robustly identified among the 93 genes potentially directly activated by Blimp-1 (66, 67). Regulatory plasma cells producing IL-10 have been reported in mice in EAE and during the *Salmonella Typhimurium* infection (3, 4) In EAE, the PB (CD138^+^ CD44^high^) subset was the main IL-10 producer population, generated through a germinal center (GC) independent pathway as the EAE course remained unchanged in Bcl6^yfp/yfp^ knock-out mice. By investigating the mechanisms by which PB produced IL-10, it was demonstrated that B cells lacking IRF4 but not Blimp-1 have an impairment of IL-10 production. The TF IRF4 binds to the *Il10* CNS-9 enhancer site in mouse PB as previously described in T cells (68). This region highly conserved in the vertebrate genome has been also described as a major site of fixation of the nuclear factor of activated T cells (NFAT1 also named NF-ATc2) (69). The involvement of different CNS in the regulation of *Il10* transcription was significantly studied in different Th cell populations. Whereas the CNS-9 seemed to be potentially active in both Th1 and Th2 lineages, the CNS+6.45 and CNS-26 are specific for Th2 cells (68), where the CNS+6.45 region is a binding site for the activator protein 1 (AP1) proteins JunB, and c-Jun. However, such distinctions are not yet clearly established in human B10 compared to other B-cell subsets. Furthermore, the TF IRF4 itself binds to the DNA in a weak-dependent manner due to its carboxy-terminal auto-inhibitory domain. IRF4 has to form a complex with AP1 family proteins like BATF or JunB to increase its binding affinity and thus regulates genes expressing the AP1–IRF consensus motif elements (AICEs) (70, 71). In B cells, IRF4 through the cooperation with BATF, JunB, and JunD was described to bound to AICEs of *Il10* and *Ebi3* (coding for an IL-35 subunit) *loci* (70, 72). In T cells, Blimp-1 expression was required in a subset of IL-10 expressing Tregs localized at mucosal sites (73). In this subset, IRF4 directly regulated Blimp-1 expression at the transcriptional level. These data were also observed in B cells following IL-21 stimulation showing that STAT3 and IRF4 cooperate to promote *Prdm1* expression (74) (**Figure 1**). More recently an elegant study investigated the IL27-driven transcriptional network highlighting several key IL-10 molecular regulators (63) suggesting that *Prdm1, Irf4*, and *Irf8* may have a suppressive role in the production of IL-10 in type 1 Tregs (Tr1). Interestingly, IRF4 and IRF8 antagonized during B-cell activation and differentiation (75) suggesting opposite roles in the control of B-cell-specific *Il10* transcription.

We thus could hypothesize that IRF4 by utilizing distinct binding partners could mediate cell-type and *stimulus*-specific IL-10 production in different lymphocyte subsets. Furthermore, the time-frame of the molecular control of IL-10 is crucial and may explain contradictory relationships. The molecular mechanisms required for the induction of *Il10* in B cells may change throughout time to ensure its maintenance leading to important modification in the cooperative binding factors. Further studies, especially kinetics studies have to be designed to identify how the core triad including BLIMP-1, IRF4 and STAT3 may cooperate or act independently in the generation of B10 cells (**Figure 1**).

### STAT3 and the NFAT Family in the Generation of IL-10 Producing B Cells

STAT3 signaling is involved in the early activation of B cells, especially in the BCR signalosome recruitment and the STAT3 deficiency led to reduction of pAkt and pErk1/2 and both mTORC1 and mTORC2 activity following BCR stimulation (76) Furthermore, STAT3 has also a non-canonical function and its localization to the endoplasmic reticulum (ER) modulates ER-mitochondria calcium (Ca^2+^) release by interacting with the Ca^2+^ channel IP3R3 (77).

The implication of the BCR signaling in the generation of B10 is still discussed. Although *in vitro* activation of splenic B cells with anti-IgM Ab failed to induce IL-10 production compared to TLR signaling, there is a dramatic decrease of B10 cell numbers in the MD4 transgenic mice with a fixed BCR rearrangement suggesting that BCR diversity may direct B10 development (43). A report using TgVH3B4 mice that express a VH derived from an actin-reactive natural Ab, 3B4 revealed that B10 cells mainly targeted self-antigens (Ag). This study also suggest that B10 could be positively selected by self Ag and that high BCR signaling could promote B10 development and IL-10 production (78). In contrast to the MD4 model where there is no virtually Ag recognition, the interaction with the Ag seems mandatory for the maintenance and generation of B10. This study is in agreement with others showing that B10 induction is negatively or positively modulated in CD19^-/-^ and CD22^-/-^ KO mice, respectively where BCR signaling is directly altered.

B cells from mice that are deficient for the ER calcium sensors stromal interaction molecules 1 and 2 (STIM1 and STIM2) have normal B-cell development but display defective activation of NFAT1 and aberrant calcium (Ca^2+^) signaling following BCR ligation (79) (**Figure 1**). In these mice, the BCR-induced dephosphorylation of NFAT1 was markedly impaired in B cells leading to a defect in IL-10 production, observations that were recapitulating with NFAT1 inhibitors such as cyclosporine or Tacrolimus (FK506). The authors further demonstrated that the double STIM1/STIM2 KO mice developed an exacerbated EAE due to a dramatic depletion of B10. These data support that a Store Operated Calcium (SOC) influx induced by BCR stimulation drove IL-10 expression in a STIM-dependent NFAT1 activation manner. Several years later, the same group demonstrated that the transfection of active calcineurin A markedly increased BCR-induced IL-10 production through activation of NFAT1 in B cells using an *Il10*-*GFP* reporter mouse model. Furthermore, they showed that this induction is repressed in *Irf4* KO mice suggesting that NFAT1-dependent IL-10 production required IRF4 and was dependent on the Ca^2+^ influx (3).

On the other hand, another report showed that mice with an NFAT2 deficiency in the B-cell compartment have two-fold more B10 cells compared with wild-type B cells and lead to the amelioration of EAE (80). More recently, similar results showed that NFAT2 ablation in B cells suppressed the induction of skin inflammation by an increase of B10 (CD1d^+^ CD5^+^ and CD138^+^ PB) in the spleen, the lymph nodes, and the blood (81) They have further shown that NFAT2 binds directly to the *Il10* gene *in vivo* and suppresses its transcription. However, the role of NFAT1 and NFAT2 in IL-10 production by B cells remains complex and differs between normal and malignant B cells. We previously demonstrated that the CD5 molecule, overexpressed in CLL B cells, promoted IL-10 expression and cell survival through a STAT3 and NFAT2-dependent pathway (82) and that IL-10 expression by B cells was directly connected with the aggressivity of the disease (83). These studies were further extended, demonstrating that NFAT2 controls multiple anergy-associated genes, BCR expression, and Ca^2+^ response in CLL B cells (84) and that knockdown of STAT3 significantly impaired the ability of CLL B cells to produce IL-10 and reverse T-cell dysfunction (85). Interestingly similar observations were made in diffuse large B-cell lymphoma (DLBCL) showing that STAT3 and NFAT2 interaction induced an immunoregulatory phenotype in malignant B cells with the upregulation of IL-10 and programmed Death-Ligand 1 (PDL1) (86).

This fine-tune regulation of Ca^2+^ signaling in B cells by its different partners including the balance between NFAT1 and NFAT2 and their interaction with STAT3 may be an important crossroad in the generation of B10 in normal and malignant B cells.

### STAT3 and Metabolic Pathways in the Generation of IL-10 Producing B Cells

**Figure 2:**
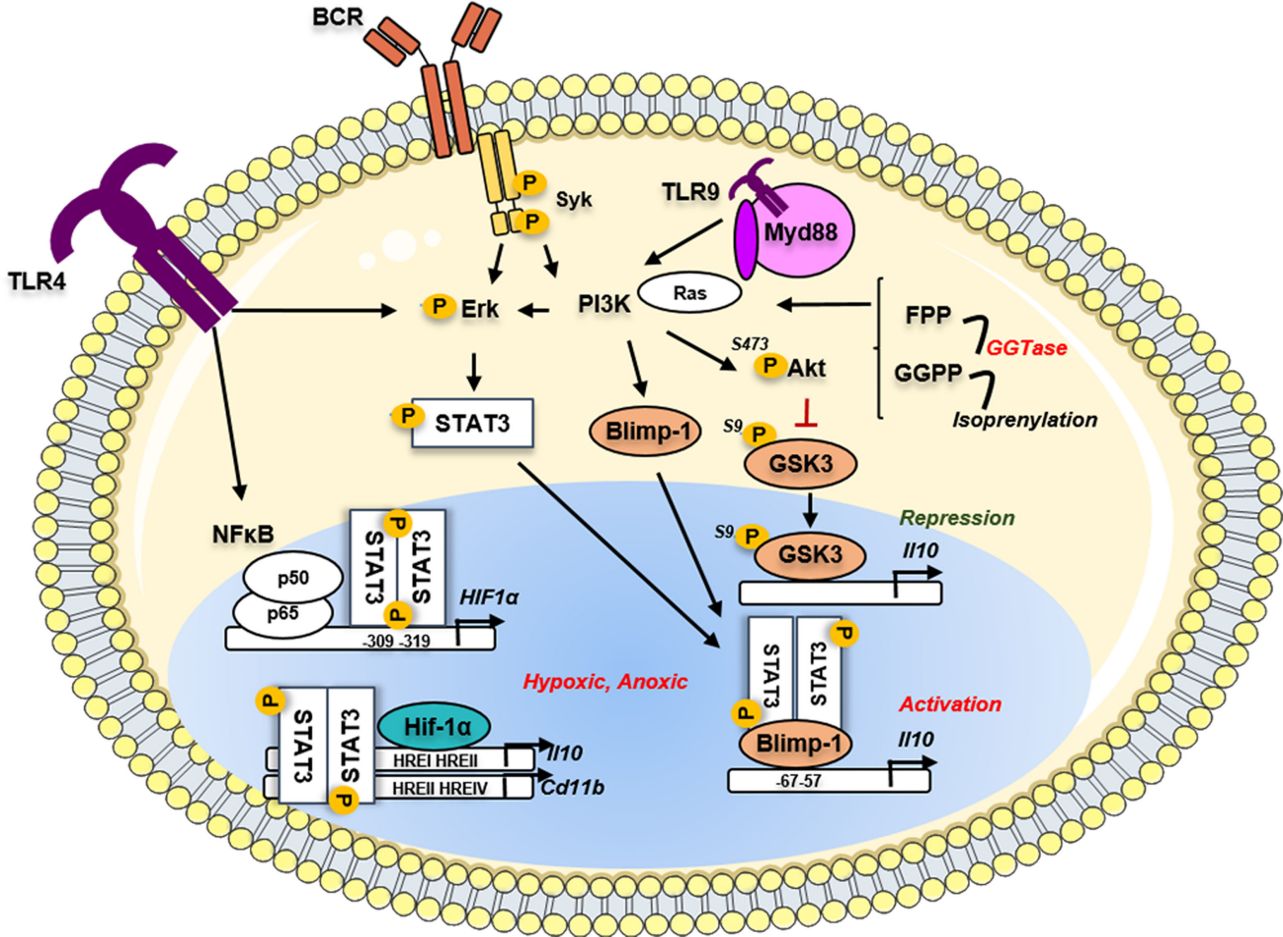
Implication of the metabolic pathways in the regulation of the *Il10* gene in Bregs. Phosphorylated STAT3 could induce metabolic pathways such as the Hypoxia sensing by the transcription of Hif-1α. The Hif1α protein binds on the HRE sites located on the *Il10* gene to induce its transcription and its production by Bregs. In parallel, another metabolic driver implicated in the multi-step transformation of acetyl-CoA to cholesterol, the geranylgeranyl transferase I (GGTi) catalyses the conversion of geranylgeranyl-PP (GGPP) to drive the isoprenylation process. This mechanism controls the Ras-dependent downstream signal of the PI3K-Akt pathway, leading to the restriction of the inhibitory GSK3 activity and thus activation of the *Il10* transcription. The GGP-dependent pathway *via* the regulation of the PI3K downstream signaling regulated the *Prdm1* gene translation and the Blimp-1 translation directly promoting *Il10* production. TLR, toll-like receptor; BCR, B-cell receptor; Myd88, Myeloid differentiation primary response protein; Syk, Spleen associated tyrosine kinase; STAT3, Signal transducer and activator of transcription 3; Blimp-1, B lymphocyte-induced maturation protein 1; PI3K, Phosphoinositide 3 Kinase; Erk, Extracellular signal-regulated kinase; GSK3, glycogen synthase kinase; Akt, Protein kinase B; Ras, Rat sarcoma virus protein; FPP, Farnesyl pyrophosphate; GGPP, Geranylgeranyl pyrophosphate; GGTase, geranylgeranyl transferase; HIF1a, hypoxia inducible factor 1 subunit alpha; NFκB, Nuclear factor kappa B; p50, p50 protein; p65, p65 protein; HRE, hypoxia response element.

The STAT3 dimers translocate to the nucleus and regulate promoter genes with STAT3-binding elements exhibiting a broad range of cellular processes (87). Among the different STAT3 responsive genes, STAT3 induces the transcription of the Hypoxia-inducible factor (HIF) 1a in BCR and LPS-activated B cells through the ERK and NFkB-dependent pathways, respectively (88, 89) (**Figure 2**). HIFs are heterodimeric TF, consisting of an oxygen-labile alpha subunit (HIF-α) and a stable beta subunit (HIF-β). Three O2-sensitive HIFα proteins have been described so far, HIF-1α, HIF-2α -also known as EPAS1, and HIF-3α (90). The B-cell-specific deletion of HIF-1α, but not HIF-2α, resulted in a defect in IL-10 production by B cells (88). Except for an important decrease of the B1a population in the peritoneum observed in Mb1^cre^Hif1a^f/f^ mice, no other defect in the B-cell maturation was observed. This group further demonstrated that in BCR-stimulated B cells, HIF-1α cooperated with pSTAT3 to bind to hypoxia-responsive element (HRE) I and HRE II regions on the *Il10* promoter under hypoxic conditions. These authors showed that HIF-1α controlled the glycolytic metabolism required for the normal expansion of CD1d^high^ CD5^+^ Bregs *in vivo*. This report was supported by an additional study demonstrating that the suppression of HIF-1α in B cells led to exacerbated colitis through CD11b and IL-10 downregulation (89). The hypoxic microenvironment has been described in the physiological situation as during the GC reaction in secondary lymphoid organs (91), but also in solid tumors, transplantation, or inflammatory diseases. The fine characterization of Bregs in normal or pathophysiological tissues promises new stimulating insights in the better comprehension of these pathophysiological mechanisms.

Beyond the control of the glycolysis pathway, another metabolic driver involved in the control of IL-10 expression in B cells was recently described implying lipid metabolism through the multi-step process of transformation of Acetyl CoA to Cholesterol. The abrogation of the HMG-CoA reductase enzyme activity by atorvastatin led to the suppression of TLR9-induced regulatory capacities of human B cells (92). It was further demonstrated that IL-10 production was dependent on the geranylgeranyl transferase (GGTase) I (GGTi) that catabolizes conversion of Geranyl-Geranyl-PP (GGPP) toward the isoprenylation pathway. By controlling the downstream Ras-dependent PI3K-AKT signaling following TLR9 stimulation, the GGTase *via* inhibitory phosphorylation on the Serine 9 prevented the activity of the glycogen synthase kinase 3 (GSK3β) and the subsequent *Il10* transcription inhibition (**Figure 2**). GSK3 was already described as an important repressor of *Il10* among the Th1, Th2, and Th17 subsets (93, 94) and in dendritic cells (95) at the epigenetic level. The comparative RNA-sequencing analysis of TLR9 stimulated B cells in the presence or absence of GGTi revealed the strong downregulation of several TF including *Prdm1, Batf* (Basic Leucine Zipper ATF-Like Transcription Factor), and the *Aryl-hydrocarbon receptor (AhR)*. Finally, the siRNA-knockdown of Blimp-1 *in vitro* led to the reduction of the TLR9-mediated IL-10 production by B cells. This study is, so far, the only report that established the direct involvement of Blimp-1 in the generation of B10 cells.

**Figure 3:**
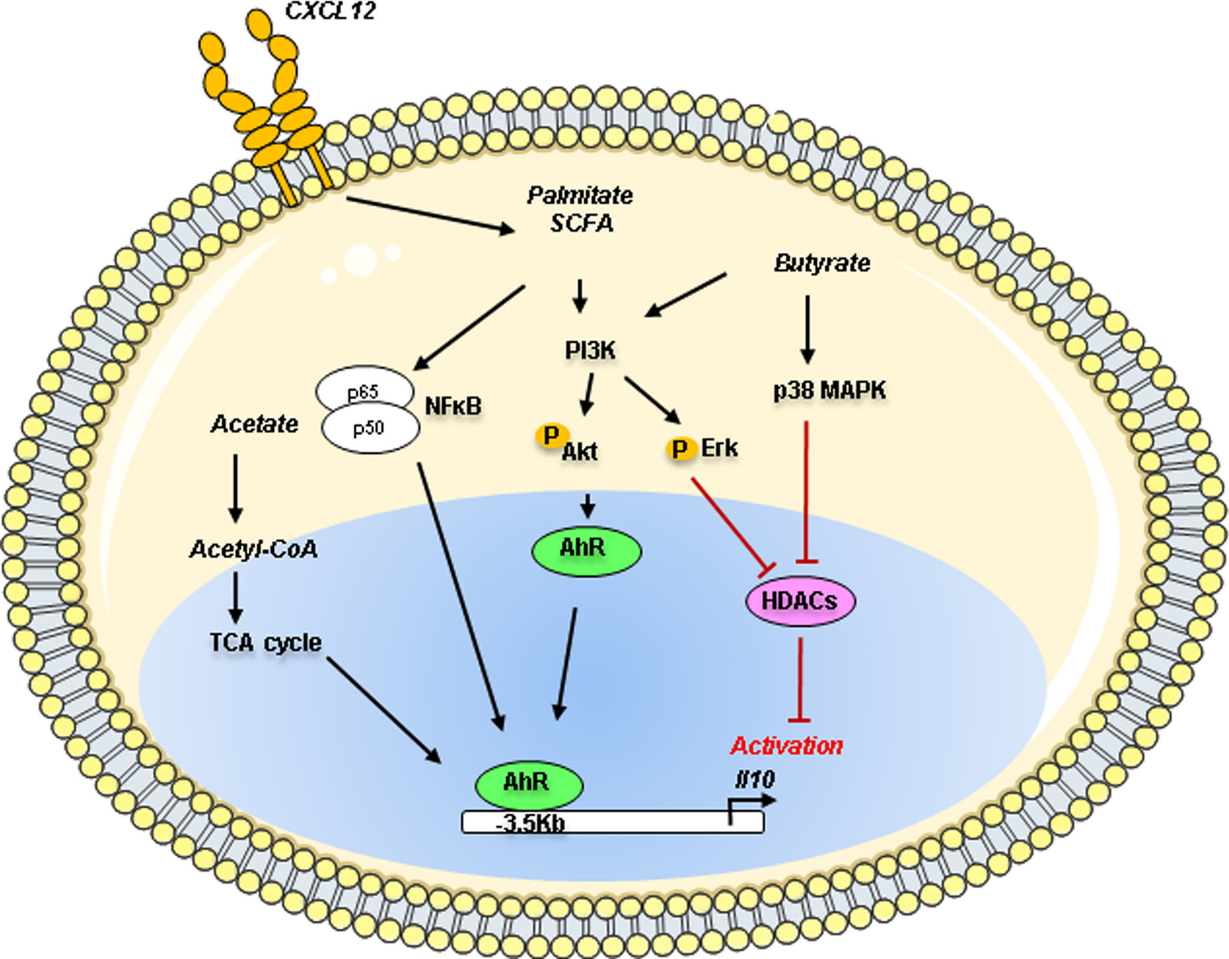
Metabolites action on the Il10 gene regulation in Bregs. Metabolites such as acetate, palmitate and butyrate can control IL-10 production in B cells through the activation of the environmental sensor Aryl-hydrocarbon receptor (AhR) known to bind on a variety of derivative metabolites. Palmitate in synergy with CXCL12 induces IL-10 production through the PI3K and NFκB pathways. Acetate through its conversion into acetyl-coA could act as a regulator of the TCA cycle, fueling the differentiation of murine B1a cells or human CD24^high^ CD27^+^ into IL-10 producing B cells (B10). Butyrate can mediate B10 expansion *via* the p38 MAPK and PI3K pathways using the histone deacetylases (HDACs). PI3K, Phosphoinositide 3 Kinase; ErK, Extracellular signal-regulated kinase; Akt, Protein kinase B; MAPK, Mitogen-activated protein kinase; NFκB, Nuclear factor kappa B; AhR, Aryl hydrocarbon receptor; TCA, tricarboxylic acid; Acetyl-CoA, Acetyl-coenzyme A; SCFA, short-chain fatty acid; HDACs, histone deacetylases; p50, p50 protein; p65, p65 protein.

In B cells, different studies have shown the AhR implication in IL-10 production in collaboration with numerous transcriptional cofactors (96) (**Figure 3**). The AhR is an environmental sensor that binds on a variety of ligands such as exogenous toxins (dioxin), xenobiotics, and endogenous derivatives metabolites from cells or microbiota. Mauri’s group has established the involvement of the AhR in the control of *Il10* transcription in the immature CD19^+^ CD21^high^ CD24^high^ B cell population in mice. *In vivo*, B cell-specific deletion of AhR caused exacerbated arthritis and promoted excessive inflammation by depleting B10. The AhR binding site seems to be located at -3.5 kb upstream of the TSS on the Il10 gene in a non-CNS region suggesting putative difference with human Bregs (97). This study was further extended by demonstrating that change in the short-chain fatty acid (SCFA) butyrate levels influenced the generation of IL10^+^ CD19^+^ CD24^high^ CD38^high^ B cells in patients with rheumatoid arthritis. By using an antigen-induced mouse model of arthritis, the authors established that the butyrate supplementation was sufficient to suppress the disease by increasing Bregs suppressive ability through an AhR dependent-manner (98) Moreover, AhR seems to also regulate the inhibitory receptor TIGIT and *Il10* expression in TIM1^+^ B cells (99). Several years before, the fatty acid palmitate was demonstrated in synergy with CXCL12 able to induce IL-10 production in adipose B cells through the PI3K and NFkB signaling (100). News studies have recently demonstrated the role of SCFA in controlling B10 generation with some discrepancies mainly due to the different *in vitro* stimulation cocktails used for the B10 induction. The SCFA acetate, known to influence the lipid and the tricarboxylic acid (TCA) cycle, was demonstrated directly promoting the differentiation of IL-10 producing B cells from B1a progenitors in mice or from CD24^high^ CD27^+^ B cells in humans with a more potent activity than the butyrate (101). The inhibition of the conversion of acetate to acetyl-CoA by the repression of acetyl-CoA synthetase member 2 (ACSS2) decreases the IL-10 production suggesting that acetate is an essential regulator fueling the energy production necessary to B10 differentiation. In another report, when B cells were pre-activated with CD40, LPS, or CpG, the addition of butyrate, and pentanoate, but not acetate, increased the percentage of B10 cells (102). They demonstrated that the molecular mechanisms of butyrate-dependent B10 expansion are independent of the mammalian G-protein-coupled receptor pair activity but acted through HDAC inhibitory action *via* a p38 MAPK and PI3K-dependent pathway. However, the precise role of histone acetylation in B10 generation remains to be established. To note, the epigenetic regulation of *Il10* expression especially in B cells is still an unresolved field of research.

Overall, these reports about metabolism control of regulator B cells have opened an exciting new area of investigation, recently reviewed by the Mauri’s group and ours (103, 104).

## C-MAF as a key driver of the B-cell immunoregulatory function?

### The Transcription Factor c-MAF

c-MAF (MAF) is a basic region-leucine zipper (bZIP) TF belonging to the AP-1 superfamily and more specifically to the large MAF family proteins. Large MAF proteins include MafA, MafB, MAF, and Nrl, which possess a transactivation domain and activate transcription by forming a homodimer. MAF contains basic regions allowing the recognition of a palindromic sequence named MAF Recognition Element (MARE) in the promoter region of target genes of which there are two types: a 13-bp T-MARE (TGCTGAG/CTCAGCA) with a TPA-Responsive Element (TRE) and a 14-bp C-MARE (TGCTGAGC/CGTCAGCA) with an AMP-Responsive Element (CRE). The MAF gene is located on chromosome 16q23.2 in humans and on chromosome 8 in mice, on the reverse strand for both (105).

**Figure 4:**
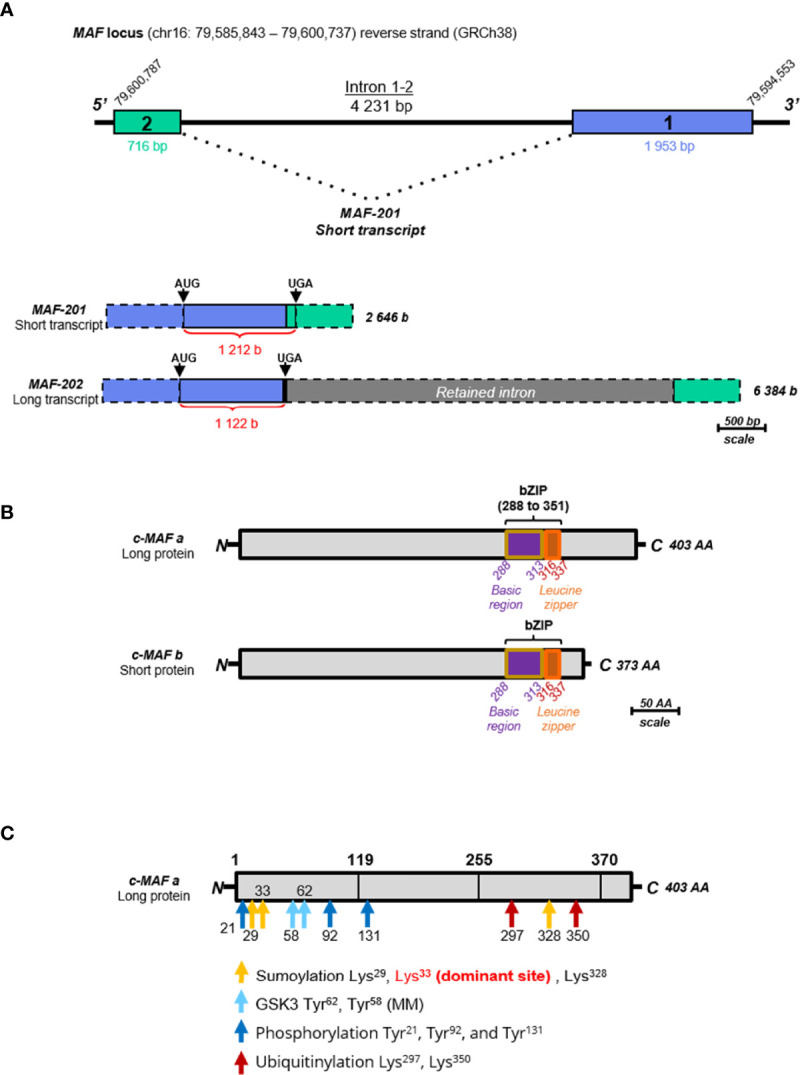
Schematic representation of the MAF gene, transcripts and proteins. **(A)** Schematic representation of the MAF gene and transcripts. The scale used is 1 cm for 500 bases (b) or base pairs (bp). The blue rectangle represents the exon 1 (1 953 bp) and the green rectangle represents the exon 2 (716 bp). The only intron (4 231 bp) is represented by a black line. The two transcripts, MAF-201 (2 646 b) and MAF-202 (6384 b) are represented below, keeping the color code of the exons. The retained intron in the long transcript MAF-202 is represented by a grey rectangle. The untranslated parts of the exons are identified by dotted borders and the length of the open reading frame of each transcript is indicated in red. These open reading frames are delimited by “start” codons (AUG) and “stop” codons (UGA). **(B)** Schematic representation of c-Maf protein isoforms. The scale used is 1 cm for 50 amino acids (AA). The long protein (c-MAF a) is composed of 403 AA and the small protein (c-MAF b) is composed of 373 AA. The entire bZIP (basic leucine zipper) domain is indicated on each isoform within violet, the basic motif of 25 AA, and in orange the leucine zipper of 21 AA. All these data are extracted from databases “Ensembl” (ensemble.org) and “Uniprot” (uniprot.org). **(C)** Schematic representation of post-translational modifications of the c-MAF proteins, using the long protein as template. Post-translational modification sites are indicated by arrows: the sumoylation sites are in yellow, GSK3 phosphorylation sites are in blue, other phosphorylation sites are in dark blue, and ubiquitination sites are in red. There are three sites of sumoylation located on the lysine 29, lysine 328, and lysine 33 which is the dominant site. There are two sites of phosphorylation by GSK3 located on tyrosines 58 and 62. Three other sites of phosphorylation are indicated in dark blue located on the tyrosines 21, 92 and 131. Two sites of ubiquitination are located on the lysines 297 and 350 and indicated in red.

Two forms of MAF mRNA have been described in humans, the variant MAF-201 referred to as the small transcript and the MAF-202 variant referred to as the long transcript. The small transcript MAF-201 (2 646 bases) was made up of the 2 exons of the MAF gene after splicing of the intron (4 231 bases) which was not spliced in the long MAF-202 transcript (**Figure 4A**). The intron retention in MAF-202 has resulted in the introduction of a premature stop codon in the open reading frame leading to the production of a short protein isoform (c-MAF b), whereas the small transcript MAF-201 encoded a longer protein isoform (c-MAF a). The c-MAF a and c-MAF b proteins from these transcripts comprised 403 and 373 amino acids, respectively. A putative third protein and mRNA (referred to as medium variant MAF-203) have been described in the *Ensembl* database, however, neither the mRNA nor the protein have been reported in experimental data. The c-MAF a protein is only found in humans whereas the c-MAF b is present in both murine and human species. The different constitutive domains of the c-MAF proteins are highly conserved between the different isoforms (**Figure 4B**). The basic domain is requisite for DNA binding while the leucine zipper allows dimerization. The EHR domain (extended homology region) is specific for the MAF proteins and is also required for MAF binding to DNA. Finally, the transactivation domain which is rich in acidic residues responsible for protein’s transcriptional activator functions is separated from the bZIP domain by a Hinge region (106).

The c-MAF proteins are subject to some post-translational modifications that could modulate their activity (**Figure 4C**). In T cells, the phosphorylation of c-MAF by the tyrosine-protein kinase Tec or by the CARMA1-dependent activation of the IκB kinase (via IKKβ) was required for c-MAF nuclear translocation and DNA binding (107–109). The serine-threonine protein kinase GSK3β-dependent c-MAF phosphorylation has been described with antagonist outcomes. GSK3β mediated c-MAF-phosphorylation and subsequent degradation through the ubiquitin-proteasome pathway in multiple myeloma. However, in presence of a coactivator complex, the phosphorylation and possibly its mono-ubiquitination may also result in the increase of its transcriptional activity (110, 111). At least nine phosphorylation sites located specifically in the activation domain of c-MAF (**Figure 4C**) were described, supporting their essential role in c-MAF activity. The SUMOylating, a post-translational modification resulting in the covalent binding of one or more SUMO proteins to a lysine motif was reported on the c-MAF protein at the lysine 33 leading to a decrease of the c-MAF transcriptional activity on the *IL-4* and *IL-21* genes (112, 113).

### MAF Regulation of the IL-10 Locus

MAF binds directly the MARE sequences of the *Il10* promoter in human macrophages (114), mouse B cells (115), Tr1 cells (96), or Th17 (116). However, several reports suggested that c-MAF could not induce *Il10* or even bound the *Il10 locus* in the absence of other co-regulatory factors like BATF or IRF1 as described in Tr1 cells (117). The protein c-MAF binds the CNS -9 together with BLIMP-1 and STAT4 and induces *Il10* in Th1 cells, but the binding of MAF is independent of BLIMP-1 expression (64). Although *Il10* represents a main target of the MAF gene, this target is far from being the only gene regulated by c-MAF. Indeed, c-MAF regulates a broad range of immune molecules as described for *RoRa, Il21, Il2, Il22* in Th17 (118), colony-stimulating factor 1 receptor (*Csf-1r*), and metabolic pathways in macrophages (119) or *Tbx21* (encoding the TF T-bet) in group 3 innate lymphoid cells (120) underlined its crucial regulatory role in the immune system (121).

### MAF Expression in IL-10 Producing T Cells and Macrophages

In T cells, extensive literature exists on the role of c-MAF and its relationship with IL-10 production [recently reviewed in (122)]. One recent elegant study underlined the context-specificity of the role of MAF in the T-cell response across different diseases model demonstrating that beyond the control of IL-10 expression, MAF acted as a negative regulator of *Il2* (123). One study has examined the role of both isoforms in the modulation of IL-10 production in human stimulated Th17 cells (116). Only the overexpression of c-MAF b (short protein) led to upregulation of IL-10 production suggesting a unique specific function of the different isoforms. Interestingly, extensive ChIP-sequencing studies revealed that c-MAF binding domains are mostly localized outside the promoters and may target enhancer sequences. Furthermore, c-MAF overexpression alone was not sufficient to convert Th17 IL-10^-^ cells into Th17 IL-10^+^ highlighting its primary role as a co-activator or co-repressor factor. The availability or competition of other cooperating factors together with the epigenetic accessibility of regulatory regions seem to be crucial for the c-MAF activity supporting its highly versatile broad range of action in many immune cells.

c-MAF is also essential during innate immunity and was first reported as a key element in macrophage polarization leading to IL-10 production and establishing the immunoregulatory M2 program (124). A recent report demonstrated that c-MAF contributes to the tumor-associated macrophages (TAMs) dampening T-cell effector function during lung cancer (119). Through ChIP-sequencing analysis, c-MAF was shown to directly regulate *Csf-1r* transcription and control important M2 markers as *Il12, Il1b*, *Il6, Arg1, Il10, Tgfb, Irf4*, and *Ccr2*. This study further demonstrated that c-MAF may act as a metabolic switch in macrophage inhibiting the glycolysis and promoting the TCA cycle and UDP-GlcNAc activity to favor macrophage conversion into immunosuppressive M2 within the tumor suggesting an important role of MAF on cell metabolic activity (119).

### MAF in B Cells

The role of MAF in B cells is very less documented and a large gap in knowledge remains regarding the intrinsic function of c-MAF in B cells. Two recent papers suggested that c-MAF could exert an activity by controlling the IL-10 production in murine and human B cells suggesting an underestimated role in Breg function.

The first study demonstrated that MAF was constitutively expressed in B220^+^ B cells isolated from the spleen and could be upregulated following LPS stimulation together with IL-10 expression (115). ChIP-PCR identified that c-MAF directly bound a MARE sequence located at the CNS-0.5 on the *Il10* gene. The knockdown of MAF using specific shRNA in LPS-stimulated B cells resulted in the decrease but not a complete abrogation of *Il10* production. A second group has more recently suggested the role of c-MAF in regulating B10 during EAE. The B-cell specific deletion of the signaling lymphocytic activating molecules (SLAM)F5 (CD84) led to an important amelioration of the disease with delayed onset and attenuate clinical score (125). This disease improvement is accompanied by an increase in the different IL-10 producing Breg populations (CD5^+^ CD1d^high^, T2-MZP, and CD138^+^ PB) in the spinal cord but also the spleen and lymph nodes. Using the Vert-x mice which express a GFP reporter on the *Il10* gene, they analyzed the transcriptome of CD19^+^ GFP^+^ cells in mice treated or not with a SLAMF5 blocking Ab revealing that SLAMF5 downmodulation increased c-MAF and AhR expression. Interestingly, these observations were also confirmed in humans showing that c-MAF is upregulated *in vitro* following SLAMF5 inhibition in total B cells. Although indirect, these results suggest that MAF could exert a key role in regulating Breg populations both in humans and mice. One interesting point is that MAF expression seems not restricted to a specific Breg subset but is broadly associated with *Il10* expression whereas AhR was demonstrated as specific to the T2/MZP subset (97). Interestingly, SLAMF5 (CD84) was also found to be downregulated in the human signature of GZMB^+^ B cells (35), suggesting maybe a broad inhibitory role in multiple Breg populations.

**Figure 5:**
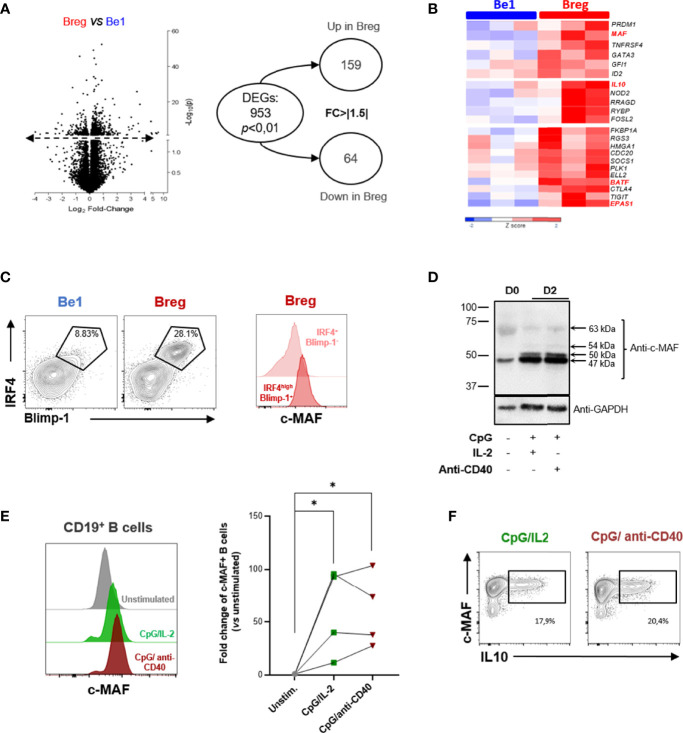
MAF is upregulated in *in vitro*-induced human B10. **(A)** Differential expressing genes between pro-inflammatory B (Be1) cells and Bregs. RNA-sequencing transcriptional analysis was performed on three pooled samples of *in vitro* induced Be1 and Bregs. Left: Volcano plot reporting p value (-log10[P], y axis) as a function of log2 fold change (FC) between samples (x axis). Transcripts that were identified as significantly differentially expressed are represented above the dotted line. On the right, the circle chart shows numbers of differentially expressed genes (DEGs) with a p value < 0.01 and a FC > ǀ1.5ǀ. **(B)** Differential expression of genes implicated in immunoregulation between Breg and Be1. Expression of genes is represented in z score with a color gradient from dark blue for down-regulated expression to red for up-regulated expression. The MAF gene highlighted in red is the transcription factor more differentially expressed between Be1 and Breg. Other genes related to immunoregulation appear upregulated in Breg compared to Be1. **(C)** Left: Assessment of the differentiation state of Be1 and Breg using IRF4 and Blimp-1 expression. Black gates indicate IRF4^high^ BLIMP-1^+^ differentiated B cells. Right: Representative histogram of MAF expression in gated differentiated IRF4^high^ BLIMP-1^+^ (dark red) and IRF4^+^ BLIMP-1^-^ (light red) cells from *in vitro* generated Bregs. The expression of MAF is higher in regulatory plasmablasts than in IRF4^+^ Blimp-1^-^ Bregs. **(D, E)** c-MAF protein expression in unstimulated or two-days stimulated B cells assessed by western blotting **(D)** or flow cytometry **(E)**. **(D)** Western Blot showing the c-MAF expression in resting unstimulated peripheral B cells (D0) and in stimulated B cells simulated (D2) by CpG with IL-2 or anti-CD40 for two days. GAPDH expression was used as an endogenous control. Black arrows indicate the calculated sizes of the protein recognized by the anti-c-MAF antibody. **(E)** Left: Representative histograms of c-MAF protein expression in unstimulated (grey) or stimulated B cells by CpG/IL-2 (green) or CpG/anti-CD40 (brown) for two days. Right: Plot representing differential expression of c-MAF between unstimulated and stimulated B cells (N = 4). The c-MAF expression is represented in fold change of unstimulated B cells. The difference of MAF expression between unstimulated and stimulated B cells have been evaluated using Kruskal Wallis test combined with Dunn’s multiple comparison test. *p-value < 0,05. **(F)** Assessment of the simultaneous expression of c-MAF and IL-10 expression production in stimulated B cells. Representative dot plots of c-MAF and IL-10 expression in the two-days stimulated B cells by CpG/IL2 (green) or CpG/anti-CD40 (brown). Black rectangles indicate the c-MAF^+^ IL10^+^ B cells. The percentage of this population is indicated below the rectangles.

We recently developed an *in vitro* model of B-cell polarization to induce pro-inflammatory B (Be1) cells and Bregs suppressing CD4^+^ T cell proliferation (126). *In vitro-*induced Breg cells included mostly activated B cells (IgM^+^ CD38^+^ CD27^-^) and unswitched PB (CD19^+^ IgM^+^ CD27^low^ CD38^+^ CD20^+^) able to simultaneously produce IL-10 and IgM. To further characterize the co-cultured B-cells, we performed RNA-sequencing (RNA-seq) transcriptional analyses on three pooled samples of B cells sorted from the two different co-culture conditions. We assessed the differential expressing genes (DEGs) between Bregs and Be1 (**Figure 5A**). We observed 953 DEGs (P-value < 0.01) and 223 genes with a FC > │1.5│ (**Figure 5A**). We underlined a significant up-regulation of genes involved in immune regulation and PB differentiation including *IL-10*, *ELL2*, *IgJ*, *PRDM1*, and the transcription factor *MAF* (**Figure 5B**). We also highlighted several key molecules that could contribute to the immunoregulatory phenotype observed *in vitro* such as BATF, FOSL2, GATA3, CTLA4, TIGIT, or EPAS1 (HIF2-α). Interestingly, FOSL2 (also called Fra-2, an IL-2 regulator), and BATF are AP1 family transcription factors that have already been described as able to interact with MAF proteins (118). We confirmed at the protein level that c-MAF is upregulated together with BLIMP-1 and IRF4 in Bregs induced in our co-culture, suggesting that in these conditions c-MAF is associated with IL-10^+^ regulatory PB (**Figure 5C**).

To examine the conditions required for c-MAF induction, we stimulated peripheral B cells with CpG and IL-2 and or anti-CD40 Ab for two days, both simulations described as potent IL-10 inductors (127, 128). Whereas c-MAF is low expressed in resting peripheral B cells, c-MAF could be induced following stimulation with an optimal response through the combination of TLR9 with IL-2 or CD40 signaling (**Figure 5D**). As previously described, the western blotting analysis revealed different sizes of proteins recognized by anti-MAF Ab suggested some post-translational modifications (107, 129). The main form (47 kDa) observed in resting peripheral B cells was preferentially increased after stimulation. Two other c-MAF isoforms of 50 and 54 kDa were absent in resting B cells but induced following stimulation. The higher form (63 kDa) could correspond to the SUMOylated c-MAF described in mice (**Figure 5D**) (112, 113). Further investigations will be necessary to elucidate the post-translational modifications and the transcripts implicated in different c-MAF proteins. We further confirmed these results by flow cytometry, with an expression 10 to 100 times higher (p-value < 0,05) compared with resting B cells (**Figure 5E**). We finally analyzed the production of IL-10 together with the c-MAF expression in stimulated B cells (**Figure 5F**). IL-10 intracellular detection was shown to be associated only with MAF-expressing B cells but was not observed in MAF^-^negative B cells. However, such observation only suggested a correlative phenomenon and further experiments are mandatory to understand the precise role of MAF on IL-10 production.

**Figure 6:**
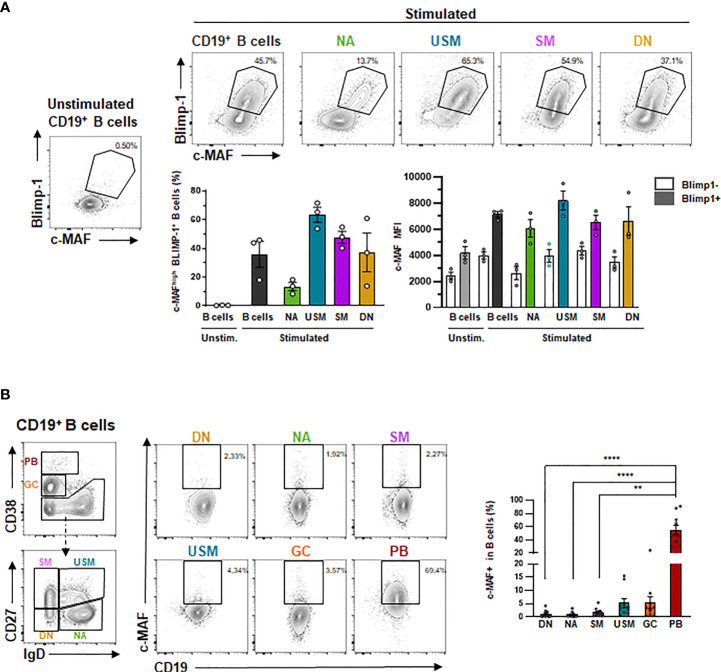
MAF is up-regulated following B-cell differentiation and expressed in human tonsils. **(A)** Expression of BLIMP-1 and c-MAF in differentiated B cells. Up: Representative expression of c-MAF and BLIMP-1 by flow cytometry in unstimulated B cells or stimulated naive (NA), unswitched memory (USM), switched memory (SM), double-negative (DN) B cells and total B cells. Black polygons indicate c-MAF^+^ BLIMP-1 ^+^ B cells. Down- Left: Percentage of MAF^+^ BLIMP-1 ^+^ B cells in unstimulated B cells (light grey) or stimulated total B cells (dark grey), naives (green), unswitched memory (blue), switched memory (purple), double-negative (yellow) B cells (N = 3). Down – Right: Mean Fluorescence intensity of c-MAF in differentiated B cells (N = 3). **(B)** Expression of MAF in the different tonsillar B-cell populations. Left: Gating strategy for defining the different of differentiated B cell subpopulations is presented. Differentiated B-cell populations are determined delimited according to the expression of CD38, CD27 and IgD in six subpopulations: double-negative cells IgD^-^ CD27^-^ (DN, yellow), naïve IgD^+^ CD27^-^ (NA, green) switched memory IgD^-^ CD27^+^ (SM, purple), unswitched memory IgD^+^ CD27^+^ (USM, blue), germinal center cells IgD^-^ CD38^high^ (GC, orange) and CD27^high^ CD38^high^ plasmablasts (PB, red). Right: Representative expression of c-MAF in the previously described subpopulations of tonsil B cells. Black rectangles indicate c-MAF^+^ B cells. The percentage of c-MAF^+^ B cells in each subpopulation is represented on the associated plot (N = 10). The difference of MAF expression between the populations have been evaluated using Kruskal Wallis test combined with Dunn’s multiple comparison test. ****p-value < 0.0001; **p-value < 0,001.

We next addressed whether MAF could be preferentially induced from a specific mature B-cell population (**Figure 6A**) and could be associated with B-cell differentiation. We thus sorted four B-cell populations according to IgD and CD27 expression and stimulated them for three days following BCR, TLR9, and IL-2 stimulation. We observed that c-MAF was upregulated in all mature B-cell subsets, however, we showed that the co-expression of c-MAF and BLIMP-1 expression is mainly observed in CD27^+^ mature differentiated B cells. Finally, as c-MAF was weakly expressed in circulating resting B-cells but could be induced following activation, we hypothesized that c-MAF could be expressed in the secondary lymphoid tissues as described in mice. We then examined MAF expression in human tonsils, showing that MAF was barely detectable in naive B cells but was found significantly increased in IgD^+^ CD27^+^ unswitched memory, GC cells, and PB(**Figure 6B**).

Although preliminary and mostly correlative, our analyses emphasized that the TF MAF is expressed in human mature B cells and could be observed in IL-10^+^ producing B cells as well as in BLIMP-1^+^ PB. We also showed that c-MAF could be physiologically observed in secondary lymphoid organs open new directions about its putative role in human B-cell homeostasis. However, further experiments are needed to fully understand its role in these different B-cell subsets. Furthermore, as described in T cells, we anticipated that MAF function will may be highly dependent on its different interacting molecular partners.

## Discussion: MAF Association With Other Regulators

**Figure 7:**
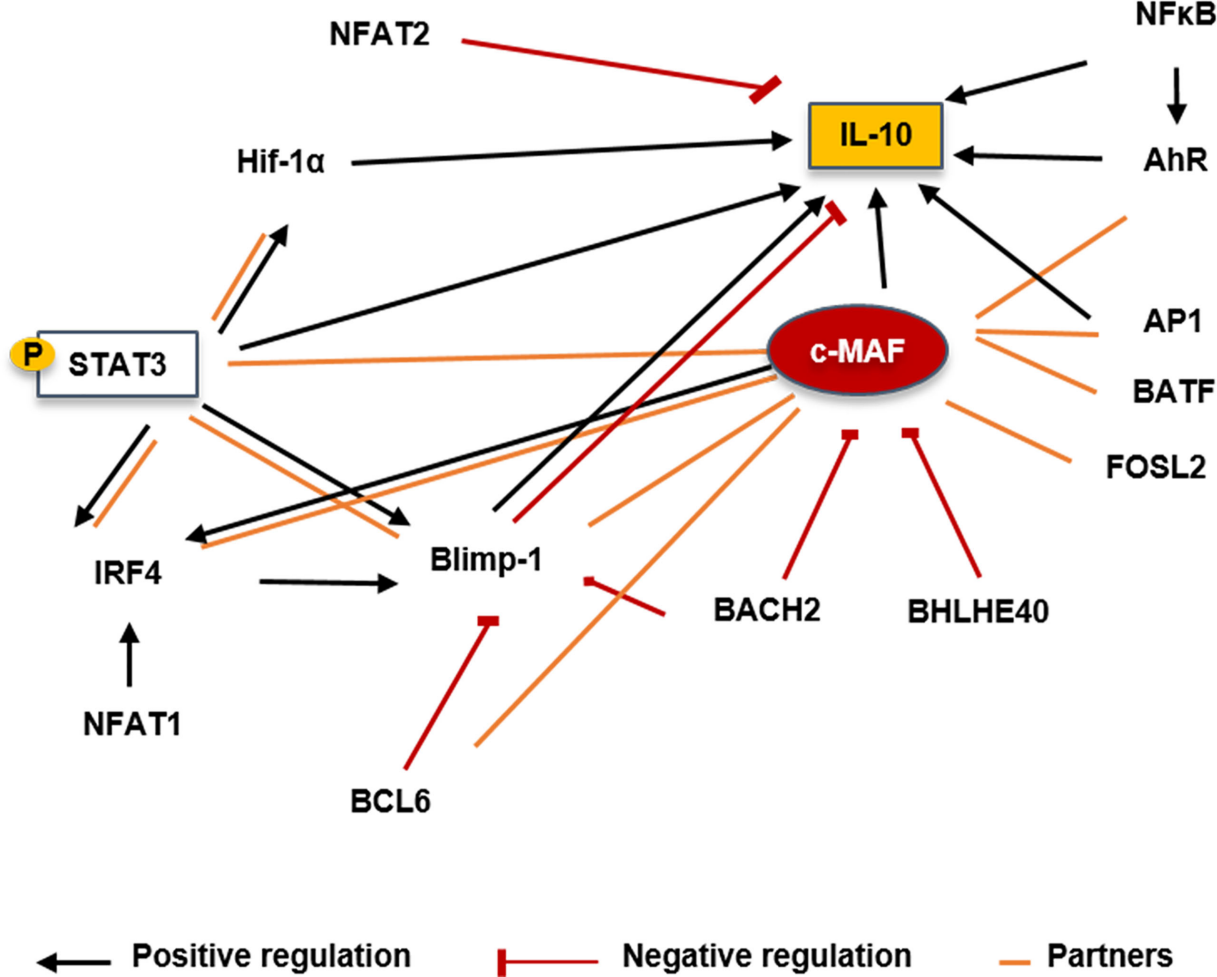
Putative molecular factors involved in controlling *Il10* transcription in Bregs. STAT3 could induce expression upon IL-6 activation and could also bind on the class II IL-6 responsive elements present on the c-MAF promoter. BACH2 has been shown to directly repress c-MAF transcription by binding its promoter in Tregs. BHLHE40 is a major repressor of IL-10 production through the inhibition of c-MAF in T cells and macrophages. BCL6 cooperates with c-MAF to regulate Tfh-associated genes. Tfh-cell differentiation and BCL6 expression is promoted through the control of c-MAF expression. IRF4 could be induced by c-MAF with the cooperation of BATF. c-MAF could directly bind on the IRF4 locus but needs a partner to transactivate its promoter. BLIMP-1 may not directly interact with c-MAF but seems to form a complex with it in T cells and both seem to have compensatory roles. Positive regulations are represented by black arrows, negative regulation by red lines and partners by orange lines. IRF, Interferon regulatory factor; Blimp-1, B lymphocyte-induced maturation protein 1; NFAT, Nuclear factor of activated T-cells; HIF1α, hypoxia inducible factor 1 subunit alpha; pSTAT3, Phosphorylated signal transducer and activator of transcription 3; AhR, Aryl hydrocarbon receptor; NFκB, Nuclear factor kappa B; AP1, Activator protein 1; IL, Interleukin; BHLHE40, basic helix-loop-helix transcription factor 40; BACH2, Broad complex-tramtrack-bric a brac and Cap’n’collar homology 2; BCL6, B-cell lymphoma 6 protein; FOSL2, Fos-related antigen 2; BATF, Basic leucine zipper activating transcription factor-like.

A study providing a highly comprehensive view of the TF network regulating Th17 has underlined the key role of MAF in adaptive immunity (118). In Th17, c-MAF was shown as a negative regulator attenuating expression of several pro-inflammatory genes such as *Rora, Runx1, Il1r1, Ccr6*, or *Tnf* while upregulating immunoregulatory other ones such as *Il10* or *Ctla4* suggesting that its role is not restricted to cytokine regulation. As a bZIP TF, c-MAF could virtually interact with all AP1 protein members. We reported as follows the MAF relationships that may be relevant in B cells (**Figure 7**).

### STAT3

STAT3 represents a key element in the transcriptional regulation of MAF expression in all helper T cells, but the mechanism remains unclear. MAF and SOX5 were demonstrated to be regulated by IL-6-mediated STAT3 activation driving Th17 expansion (130). Moreover, STAT3 binding on class II IL-6 responsive elements was observed on the MAF promoter in Th2 cells after TCR and IL-6 activation (131). This observation was also reproduced in CD69^+^ FOXP3^+^ Tregs where the treatment of cells with STAT3 and STAT5 siRNA reduced MAF expression (132). The TGFβ seems to synergically act with IL-6 to induce MAF expression by sustaining the STAT3 expression (133). IL-21 and IL-6 are upstream inducers of STAT3 activation and highly involved in plasma cell differentiation suggesting that they could also act in inducing MAF in B cells (134). Moreover, dysregulated STAT3 signaling has been reported in B cells in autoimmune diseases associated with abnormal GC reaction or plasma cell expansion (135). Does STAT3 influence B10 generation by regulating MAF expression? It is an ongoing question and several reports have already suggested that STAT3 deficient signaling contributes to impair IL-10 production in SLE B cells (48, 53).

### BACH2

BACH2 is a TF involved in B cell differentiation and is controlled in human B cells by the IL-2 signaling (136, 137). BACH2 was mostly described as able to form heterodimers with small c-MAF proteins (136) binding to MARE domains. BACH2 interacts with MAFK to negatively regulate the immunoglobulin heavy chain gene 3’ enhancer and the PRDM1 gene (138) controlling B cell differentiation and isotype switching. The BACH2 and the large MAF proteins interaction was not reported in B cells but BACH2 deletion in T cells led to an increase of the generation of Tfh-producing IL-4 and subsequent generated humoral autoimmunity (139). In this study, BACH2 was shown to directly repress *MAF* transcription by binding its promoter. BACH2 inhibits the differentiation of mature Tregs by competition with IRF4-recruiting AP-1 complexes for MARE genomic binding sites resulting in the decrease of Treg-associated genes such as *Il10, Tigit*, and, *Maf*. Interestingly, our previous data have shown that B-cell differentiation is associated with an upregulation of a similar pattern of genes suggesting that BACH2 could also repress *MAF* and *IRF4* expression in B cells.

### BHLHE40

The basic helix-loop-helix transcription factor 40 (Bhlhe40) is a member of the basic helix-loop-helix transcription factor family. BHLHE40 is a central mediator of both inflammation and pathogen control in T cells and macrophages (review in (140)). BHLHE40 was found as a major repressor of *Il10* in IL-27 driven Tr1 or Th1 cells through the inhibition of MAF (63, 141). During *Mycobacterium tuberculosis* infection, BHLHE40 was shown to bind directly to the +6kb (CNS3) enhancer of the *Il10 locus* in T cells and myeloid cells (142). BHLHE40 also named DEC1, STRA13, or Clast5, was first described as a negative regulator of B-cell activation and is downregulated following LPS or BCR stimulation (143). BHLHE40 overexpression led to cell cycle arrest in WEHI231 B lymphoma cells. By studying Bhlhe41^–/–^ mice, and DKO Bhlhe41^–/–^ Bhlhe40^–/–^, it was demonstrated that compared to BHLHE41, BHLHE40 contributes slightly to B1 development and it is still not clear whether BHLHE40 possesses intrinsic action on murine B-cell development (144). However, it was shown that BHLHE40 was induced in human B cells following BCR or TLR9 stimulation restraining proliferating capacities (145). Moreover, BHLHE40 is overexpressed in anergic CD21^low^ B cells of patients with hepatitis C virus, a subset recently underlined as pathogenic in autoimmune diseases. The role of BHLHE40 in controlling Breg expansion has to be determined and interestingly, we recently underlined that BHLHE40 is a common factor downregulated during the PB differentiation (67).

### BCL6

The BCL6 protein was first described as a master regulator of B cells during the GC reaction (146, 147). There exists a reciprocal regulatory loop between Blimp-1 and BCL6 during the B-cell differentiation, whereby both TF antagonize the expression of each other (148, 149). This antagonism between BCL6 and BLIMP-1 was also described as a critical mechanism regulating the development of Tfh (150). BCL6 and c-MAF cooperate to regulate the expression of Tfh-associated genes like *PD1* or *CXCR5* (151). Furthermore, T cells lacking MAF expression failed to develop into Tfh after immunization (152). More recently the regulatory circuit between c-MAF, BLIMP-1, and BCL6 during the Tfh differentiation was examined in *Thpok* (encoded by the Zbtb7b gene) deficient mice (153). *Thpok* promoted Tfh cell differentiation and *Bcl6* expression through controlling *MAF* expression but independently of the repression of BLIMP-1.

### IRF4

IRF4 is a key factor in the induction of regulatory plasma cells. Through a multi-layer -omics approach, c-MAF was suggested to interact with IRF4 in normal and myeloma plasma cells although no experimental data supported this idea (154). In T cells BATF and c-MAF cooperated to induce IRF4, to promote IL-4 production, and it was observed that c-MAF could bind to the IRF4 locus although it could not transactivate the promoter alone (155). To note, the action of c-MAF and IRF4 was also described in the M12 B cell lymphoma line acting in synergy with NFAT1 to enhance the IL-4 transcriptional activity (156)

### BLIMP-1

The relationship between *MAF* and *PRDM1* has been studied with attention in Th1 and Th17. Although both factors seem to be required for *Il10* expression in both subsets, there are contradictory reports about their putative interaction as previously discussed (64). Whereas some reports suggested no physical interaction between both TFs, another study using co-immunoprecipitation assay suggested that MAF, Blimp-1, and RORγt form a complex in IL-27 stimulated T cells (157)

Moreover, a study that recently examined the regulatory network involved in the control of T-cell exhaustion in tumors underlined that although *MAF* and *PRDM1* are together involved in controlling several co-inhibitory receptors expression in T cells, both factors do not interact together and that *PRDM1* function in tumor-infiltrating T cells is independent of MAF suggesting that both TFs may have a compensatory role (116) Indeed, regression of tumor was observed more significantly in *MAF*, *PRDM1* double KO mice than in mice with a single gene deletion. Our data reported an association of *MAF* and *PRDM1* during the B cell differentiation, thus a role of MAF in the B-cell central regulatory network involving *BACH2*, *BCL6*, *IRF4*, and *PRDM1* is likely to also operate in the B cell differentiation and need to be further examined.

### Role of the MicroRNAs miR155 and miR 21

MicroRNAs (miRNAs) are small non-coding RNA molecules that control gene expression by binding messenger RNA (mRNA). Whereas most of the studies demonstrated that the binding of miRNAs to 5′ UTR and coding regions have silencing effects, some others have also suggested that miRNA interaction with promoter region could have a positive effect and induce transcription (158). Of particular interest in B-cell physiology are the miR-155 and the miR-21.

First observations in mir155-deficient mice suggested that miR-155 control the GC formation, as well as, the extrafollicular response (159). It was further demonstrated that one main target of the miR155 was a member of the Ets domain-transcription factor family the TF PU.1 (160). Interestingly, the deletion of miR155 led to the overexpression of PU.1 resulting in the impairment of class switch recombination and plasma cell formation mostly by controlling the TF Pax5 (161).

The failure to downregulate Pax5 in PU.1-overexpressing cells prevented them from upregulating Blimp1 and thus initiating the plasma cell differentiation program. However, no direct role of miR155 on Blimp1 has been established so far.

The role of miRNA on IL-10 production is not clear. A recent report suggested that miR155 increased the expression of IL-10 by both upregulating the STAT3 phosphorylation and repressing the epigenetic regulator Jarid2 leading to the decrease in histone H3 lysine 27 trimethylation of the IL-10 promoter (162). On the other hand, the IL-10 production following LPS stimulation could inhibit miR155 in B cells in a STAT3-dependent manner (163). These complex interactions between IL-10, mir155, and STAT3 at the crossroad between B-cell differentiation and B-cell regulatory function need to be further elucidated but underlined their close relationship.

In one seminal paper, MAF has been described as an important target of miR-155 in T cells, and the increase of the Th2 profile observed in the miR-155 KO mice (bic^m1/m1^ and bic^m2/m2^) was partially attributed to the upregulation of the MAF protein in absence of its antagonist (164). The miR-155 suppressed MAF expression in microglia leading to the decrease of IL-10 thus promoting neuroinflammation (165). In Group 2 innate lymphoid cells (ILC2), miR-155 targets the 3′-UTR of the Maf mRNA and increased the production of IL-5, IL-9, and IL-13 Th2 cytokines in culture. Moreover, the treatment of mice with induced-allergic rhinitis with miR-155 antagomir significantly increased the expression of c-Maf and reduced allergic symptoms (166). Furthermore, another report described that c-MAF binding to the *Il10* promoter is restricted by *jarid2* in miR-155-deficient Th17 cells, emphasizing the importance of this network in regulating IL-10 expression.

Considering the key role of miR-155 in controlling the GC reaction, PB differentiation, IL-10 production, and MAF expression, we could anticipate that its expression may highly vary throughout the B-cell maturation. An in-depth investigation of these interactions could decipher important regulator mechanisms of the B-cell effector function.

The miR-21 was also described as a critical regulator of the B-cell differentiation and IL-10 production. Analysis of miR-21 levels in B cells revealed higher expression in activated B cells, especially in GC and memory B cells, and decreased expression in plasma cell differentiation (167). BLIMP-1 binds to the promoter of the pre-miR-21 to repress its expression upregulating miR-21 target genes, BTG2, PDCD4, and RHOB.

The miR-21 downregulated IL-10 expression in LPS-stimulated B cells *in vitro* and its expression is reduced in CD1d^hi^CD5^+^ and Tim-1^+^ regulatory B cells. Furthermore, the administration of miR-21 antagonist *in vivo* decreased neuroinflammation and ameliorated the clinical symptoms of the EAE mostly through the increase of IL-10 producing B cells (168). However, the fine-tune mechanisms of miR-21 regulating IL-10 expression appear more elaborate, as one of its main targets the PDCD4 protein was also described as a potent IL-10 inhibitor (169). Interestingly, PDCD4 controls MAF expression by sequestering the Basic helix-loop-helix (bHLH) transcription factors Twist2 from the MAF promoter (170).

The list of miRNAs that could potentially control IL-10 producing B cells described here is far from being exhaustive, and it is not clear how the interplay between these different actors could influence the B-cell function. However, this entire field remains an exciting area of investigation still uncovered.

## Concluding Remarks

The idea of a unique TF regulating all Breg populations seems unlikely, the diversity of precursors and mechanisms of action of Bregs are more compatible with microenvironmental plasticity and multiple molecular mechanisms. However, the extreme flexibility of the c-MAFs proteins to directly or indirectly cooperate with diverse partners modulating cell specification makes it a promising candidate in regulating B-cell effector functions such as Ab production or immune regulation.

## Material and Methods

### Patients and Samples

The study was performed in accordance with the Declaration of Helsinki and was approved by ethical committees. The patients/participants provided their written informed consent to participate in this study.

### RNA Sequencing Sample Preparation

The *in-vitro*-induced Be1 and Be2/Breg were obtained in coculture with naive and memory T cells, respectively, as previously described (126). After coculture, B cells were sorted with the “FACS MoFlo XDP cell sorter” (Beckman Coulter) with a purity greater than 98%.

### RNA-Sequencing

RNA-seq (50 bp paired-end) was performed on a Nextseq500 instrument (Illumina). The library preparation was done using the Illumina TruSeq^®^ Stranded mRNA Sample preparation kit. The starting material (1 µg) of total RNA was mRNA enriched using the oligodT bead system. The isolated mRNA was subsequently digested using enzymatic fragmentation. Then, the first strand and second strand synthesis were performed and purified (AMPure XP, Beckman Coulter). Next, the double-stranded cDNA was end repaired, 3’ adenylated, and Illumina sequencing adaptors were ligated onto the fragment ends. Finally, purified mRNA-stranded libraries were pre-amplified by PCR and the library size distributions were validated and quality inspected on a Bioanalyzer (high sensitivity DNA chip). High quality libraries were quantified using qPCR, the concentration was normalized, and the 12 samples from six independent experiments were randomly pooled in six final libraries, three for the Be1 conditions and three for the B reg conditions. The library pools were re-quantified with qPCR and the optimal concentration of the library pools was used to generate the clusters on the surface of a flow cell before sequencing on a Nextseq500 instrument using a High Output sequencing kit (2 x 50 cycles) according to the manufacturer’s instructions (Illumina). The quality of the Raw.fastq files was analyzed using the bcl2fastq software (Illumina), with a Q-score greater than 30. Reads mapping to the reference genome (GRCh37/hg19) were performed on quality-checked reads using STAR 2.4.1c. The reference annotation used was Ensembl_75. The overlap of reads with annotation features found in the reference *gtf* file was calculated using featureCount. The output computed for each sample (raw read counts) was then used as the input for Edge R analysis. Genes that had an expression level of under 10 counts per million (cpm) were excluded from the analysis. The quantile-adjusted conditional maximum likelihood (qCML) method was used to determine differentially expressed genes (DEGs) with p-value filters <0,01 and |fold-change (FC)|≥1,5.

### Peripheral Blood B-Cell Isolation, Sorting, and Culture

The leukoreduction system chambers (LRSC) of Peripheral blood from healthy donors were obtained during routine plateletpheresis at the “Etablissement français du sang”. The peripheral Blood Mononuclear Cells (PBMCs) were isolated using density gradient centrifugation on lymphocyte separation medium, Pancoll human (PAN Biotech). CD19^+^ B cells were purified from human PBMCs using the REAlease^®^ CD19 Microbead Kit (Miltenyi Biotec) with a purity greater than 98%, according to the manufacturer’s instructions.

Isolated B cells were cultured in 96-well plates (BD Falcon) at 2x10^5^ cells per 200 µL/wells in complete medium (RPMI 1640 medium (Sigma-Aldrich)) supplemented with 10% of heat-inactivated Fetal Calf Serum (FCS) (BD Biosciences), 2 mM Glutamax (Gibco) and penicillin (200 U/mL) and streptomycin (100 µg/mL). B cells were stimulated for 48 h (2 days) in the presence of CpG oligodeoxynucleotide (ODN-2006) (1 µM; *In vivo*Gen), recombinant human Interleukin-2 (rh IL-2) (20 ng/mL, ImmunoTools) or anti-CD40 (1 mg/mL) (MAB-59; Beckman Coulter).

For the sorting of B cell subsets, isolated B cells were stained for 30 min at 4°C into the dark with IgD (IA6-2, Biolegend), CD10 (ALB1, Beckman coulter), CD27 (1A4CD27, Beckman coulter), CD19 (J3-119, Beckman coulter) Abs. CD10-positive B cells, which correspond to the transitional subset, were excluded from the cell sorting. Naive (NA, IgD^+^ CD27^-^ CD10^-^), unswitched memory (USM, IgD^+^ CD27^+^ CD10^-^), switched memory (SM, IgD^-^ CD27^+^ CD10^-^) and double negative B cells (DN, IgD^-^ CD27^-^ CD10^-^) were sorted. Sorted cells were cultured in 96-well plates (Falcon) at 2×10^5^ cells per 200 µL/well in complete medium (RPMI 1640 medium supplemented with 10% of heat-inactivated FCS, 4 mM L-Glutamine (Gibco) and penicillin and streptomycin. B cell subsets were stimulated for 84 h (3.5 days), in the presence of the AffiniPure Goat anti-Human IgG+IgM (H+L) (2 µg/mL), the AffiniPure F(ab’)2 Fragment Goat Anti-Human Serum IgA, α Chain Specific (2 µg/mL; Jackson ImmunoReasearch Laboratories), CpG oligodeoxynucleotide (ODN-2006) (0,25 µM), recombinant human Interleukin-2 (rh IL-2) (20 ng/mL), as previously described (https://www.biorxiv.org/content/10.1101/2021.03.31.437810v1).

### Isolation of Tonsil B Cells

Tonsils were collected during tonsillectomy. The Tonsillar Mononuclear Cells (TMCs) were isolated by mechanical disruption of tonsil tissue followed by Pancoll gradient centrifugation. Isolated TMCs were diluted in FCS up to a concentration of 10.10^6^ TMCs/mL. Sheep red blood cells (Boehringer Ingelheim Therapeutics) were pre-incubated with Alserver solution (114 mM of dextrose, 27 mM of sodium citrate, 71 mM of sodium chloride, 1 M of citric acid diluted in distilled water) for 5 minutes and washed before an incubation at 37°C with 1 U/mL of neuraminidase (Sigma-aldrich) for 1 h. Neuraminidase-treated sheep red blood cells were washed and then incubated with the diluted TMCs during 10 minutes at 37°C. B cells from TMCs were then isolated following Pancoll gradient centrifugation, with a purity greater than 96%.

### Flow Cytometry

All staining was performed using the Navios II flow cytometer (Beckman Coulter). The Abs with the following specificities were used: CD19 (J3-119), CD38 (LS198-4-3), CD27 (1A4CD27), IgD (IA6-2) c-MAF (T54-853, BD Biosciences), BLIMP-1 (646702, R&D systems) and IRF4 (REA201, Miltenyi Biotec), IL-10 (JES3-9D7, Biolegend). Intranuclear staining of IRF4, BLIMP-1 and c-MAF (T54-853, BD Biosciences) were performed using the Transcription Factor Buffer Set according to the manufacturer’s instructions (BD Biosciences). For the detection of IL10 and c-MAF together, we used the CytoFast Perm Fix Set (BioLegend) according to the manufacturer’s instructions. The c-MAF expression was analyzed using an unconjugated rabbit anti-MAF (BLR045F, Bethyl laboratories) revealed by a Fluorescein (FITC) AffiniPure F(ab’) ₂ Fragment Donkey Anti-Rabbit IgG (H+L) (Jackson ImmunoResearch Laboratories). Flow cytometry data were analyzed using Kaluza Analysis (v2.1) and FlowJo (v10.7.2) softwares.

### Western Blot

Total proteins were extracted with RIPA Lysis Buffer (Thermo Fisher Scientific) supplemented with 1% of protease inhibitor cocktail (Sigma Aldrich). Samples treated with reducing Laemmli buffer (1.8% β-mercaptoethanol; Sigma Aldrich) were separated on SDS-PAGE with 10% acrylamide/bis (37,5:1) solution (Biorad) with the Mini-PROTEAN^®^ Tetra Vertical Electrophoresis Cell (Biorad) at 120 volts for 20 minutes and then at 160 volts for 45 minutes. Separated proteins were then transferred to a 0,2 µm PVDF membrane (Biorad) using the Trans-Blot^®^ SD Semi-Dry Transfer Cell (Biorad) at 20 volts during 90 min. After washing in Tris Buffered Saline 0,1% Tween (TBST), the membrane was blocked for 1 hour in TBST containing 5% of fat dry milk (for c-MAF) or 5% of BSA (for GAPDH) at room temperature. The polyclonal rabbit c-MAF Ab (ProteinTech) at 0,7 µg/mL in TBST containing 1% of fat dry milk and the membrane was incubated in this solution for 2 hours at room temperature. The secondary antibody used was a peroxydase conjugated donkey anti-rabbit IgG (H+L) Ab (GE Healthcare) at 0,2 µg/mL in 1% of fat dry milk TBST during one hour at room temperature. The HRP-Rabbit polyclonal to GAPDH (Abcam) in TBST containing 1% of BSA and the membrane was incubated overnight. The PVDF membranes were revealed by chemiluminescence (Immobilon Fort Western HRP substrate, Merck Millipore) by using the ChemiDoc™ XRS system (Biorad, Quantity One^®^ Software).

### Statistics

All statistics and graph representation were performed with GraphPad Prism (v.8.8.2). The Kruskal Wallis test combined with Dunn’s multiple comparison test was used.

## Data Availability Statement

The datasets presented in this study can be found in online repositories. The names of the repository/repositories and accession number(s) can be found below: https://www.ncbi.nlm.nih.gov/geo/, GSE112448.

## Ethics Statement

The study was performed in accordance with the Declaration of Helsinki and was approved by the Brest Hospital ethical committee. Written informed consent for participation was not required for this study in accordance with the national legislation and the institutional requirements.

## Author Contributions

LLP and SH designed the study. MB, MM-C, and LLP wrote the first draft of the manuscript and figures. AG, MB, MM-C, and QS performed and analyzed the experiments. SH wrote the final version of the manuscript. DC, J-OP, and OM participated in the text edition. All authors contributed to the article and approved the submitted version.

## Funding

This work has been carried out thanks to the support of the LabEx IGO program (n° ANR-11-LABX-0016-01) funded by the «Investissements d’Avenir» French Government program, managed by the French National Research Agency (ANR).

## Conflict of Interest

The authors declare that the research was conducted in the absence of any commercial or financial relationships that could be construed as a potential conflict of interest.

## Publisher’s Note

All claims expressed in this article are solely those of the authors and do not necessarily represent those of their affiliated organizations, or those of the publisher, the editors and the reviewers. Any product that may be evaluated in this article, or claim that may be made by its manufacturer, is not guaranteed or endorsed by the publisher.
